# A comparative study on prognostic differences between men and women with non-small cell lung cancer across different antitumor treatment modalities

**DOI:** 10.1186/s12885-025-15179-5

**Published:** 2025-11-21

**Authors:** Feiyang Li, Fang Li, Haowei Lu, Dong Zhao

**Affiliations:** 1 Department of Medical Oncology, Lixin People’s Hospital of Bozhou City, Bozhou, Anhui Lixin 236700 China; 2 Department of Medical Oncology, Affiliated Hospital of Qinghai University, Xining , Qinghai China

**Keywords:** Non-Small cell lung cancer, Gender differences, Prognosis, Antitumor treatment, Propensity score matching

## Abstract

**Objective:**

This study aims to investigate potential prognostic differences between male and female patients with non-small cell lung cancer under various antitumor treatment modalities, as well as to analyze potential factors influencing these differences.

**Methods:**

This retrospective study included data from 715 non-small cell lung cancer patients who were diagnosed pathologically at our center between 2018 and 2024. The dataset comprised demographic characteristics, tumor features, and treatment-related information. Propensity score matching was employed to adjust for baseline imbalances between male and female groups. Prognostic differences based on gender were assessed using Kaplan-Meier survival analysis, log-rank tests, and multivariate Cox regression models, with additional subgroup analysis.

**Results:**

Following propensity score matching, the median survival for male patients was 23 months, while female patients had a median survival of 29 months. The mortality risk for males was 29.2% higher compared to females (HR = 1.292, 95% CI 1.003–1.664, *P* = 0.044). In the EGFR-TKI subgroup, the mortality risk for males was 49% higher than for females (HR = 1.490, 95% CI 1.035–2.145, *P* = 0.028), whereas no significant gender-related differences were observed in the immune checkpoint inhibitor subgroup. Multivariate Cox regression analysis further confirmed that male patients faced a significantly increased mortality risk compared to females (HR = 1.31, 95% CI 1.01–1.70, *P* = 0.046). Furthermore, AJCC T stage, N stage, and M stage were identified as independent prognostic factors. Chemotherapy and anti-angiogenesis therapy were associated with a reduced mortality risk. Subgroup analysis revealed that male patients had significantly worse prognoses than female patients, particularly among those with early lymph node metastasis (N0-1), no distant metastasis (M0), no brain metastasis, and those who underwent surgery.

**Conclusion:**

Gender is an independent prognostic factor in NSCLC. Male patients generally experience shorter overall survival compared to female patients, particularly in those receiving EGFR-TKI therapy and in certain clinical stages. Gender should be considered in clinical practice when making treatment decisions. Future multicenter prospective studies are needed to further validate these findings and explore the underlying biological mechanisms.

## Introduction

According to GLOBOCAN 2022 data, approximately 2.48 million new cases of lung cancer were reported globally, representing 12.4% of all cancer cases, making lung cancer the most prevalent cancer type. Additionally, lung cancer remains the leading cause of cancer-related deaths worldwide. In 2022, an estimated 1.8 million lung cancer deaths occurred, accounting for 18.7% of all cancer-related deaths globally [1, 2]. Lung cancer is primarily classified into two pathological types: non-small cell lung cancer (NSCLC) and small cell lung cancer, with NSCLC being the most common, comprising approximately 85% of all lung cancer cases [3]. According to data from the Surveillance, Epidemiology, and End Results(SEER) database in the United States, the 5-year relative survival rate for all stages of NSCLC is 26%, with survival rates for patients in stage III and stage IV both below 15%. While advancements in therapies such as molecular targeted therapy and immune checkpoint inhibitors(ICIs) have improved survival rates for patients with advanced-stage NSCLC, the disease continues to represent a major public health challenge [4].

NSCLC is a highly heterogeneous malignancy. Recent studies have increasingly highlighted significant gender-related differences in disease characteristics, treatment toxicity, and survival outcomes, influenced by the interplay of biological factors, hormonal levels, and environmental exposures [5, 6]. However, research on the role of gender in NSCLC has historically been delayed due to the National Institutes of Health’s (NIH) stricter inclusion criteria for women in clinical trials, aimed at protecting women of childbearing age from potential risks. It was only in October 2014 that the NIH implemented a policy mandating the use of gender-balanced analysis methods in research [7, 8]. This delay may have contributed to a limited understanding of treatment outcomes in NSCLC, leading to disparities in survival rates and treatment-related toxicity among different patient groups.

In recent years, the treatment landscape for NSCLC has undergone significant changes with the widespread adoption of novel therapies, including molecular targeted therapy, ICIs, and anti-angiogenesis targeted(AAT) therapy. The introduction of these innovative therapeutic strategies may further amplify the influence of gender on NSCLC, leading to more complex effects on patient survival outcomes [9–11]. However, despite the growing importance of this issue, research on the impact of gender differences across various treatment modalities remains limited, which underscores the need for this study. This study aims to leverage real-world data to explore survival differences between male and female NSCLC patients, considering varying tumor characteristics and multimodal treatments. The findings will not only enhance the understanding of gender-related effects on NSCLC but also inform the development of more personalized treatment strategies for clinical practice and serve as a reference for the design of future phase III prospective studies.

## Methods and data sources

### Data sources and patient selection

**Fig. 1:**
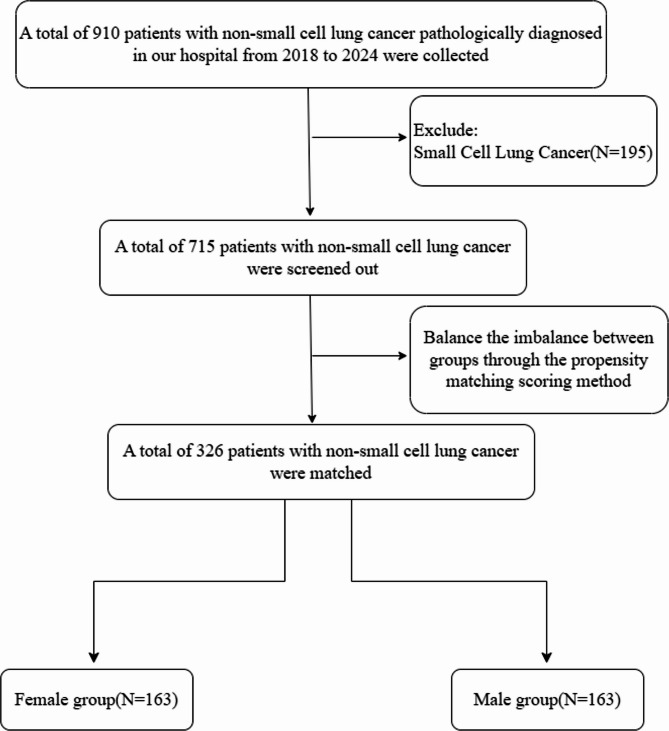
Screening Patient Flowchart

This study retrospectively collected data from patients diagnosed with malignant lung tumors at our center between 2018 and 2024, including demographic characteristics, tumor-related data, and survival information. The study was conducted in strict adherence to the 2024 revised version of the Declaration of Helsinki. To ensure the quality of the study, predefined inclusion and exclusion criteria were applied to filter the data. Inclusion criteria: (1) Pathological diagnosis of NSCLC, including squamous cell carcinoma and adenocarcinoma; (2) Patients who have undergone antitumor treatment. Exclusion criterion: Patients with missing key information. The specific screening process is illustrated in Fig. 1.

### Data collection

The data collected included the following variables: age at diagnosis (< 70 years, ≥ 70 years), gender (male, female), tumor location (left lung, right lung), T stage (T1, T2, T3, T4), N stage (N0, N1, N2, N3), M stage (M0, M1), distant metastasis (bone, brain, liver, lung), pathological type (squamous cell carcinoma, adenocarcinoma), Epidermal Growth Factor Receptor(EGFR) status (positive or negative), surgical treatment (yes or no), chemotherapy (yes or no), radiotherapy (yes or no), AAT therapy (yes or no), EGFR Tyrosine Kinase Inhibitor(TKI) treatment (yes or no), ICIs treatment (yes or no), overall survival(OS) time, and survival status.

### Statistical analysis

This study performed descriptive statistical analysis on patient data, with categorical variables presented as frequencies and percentages. The Chi-square test was used to compare demographic and tumor characteristics between male and female patient groups. To correct for baseline imbalances between the two groups and control for potential confounding factors, propensity score matching(PSM) was applied to pair patients from both groups. After matching, stratification based on gender, EGFR-TKI treatment, and ICIs treatment was performed, and Kaplan-Meier survival curves were plotted to illustrate the OS rate at each time point during follow-up. Log-rank tests were employed to compare survival differences between the two groups. Additionally, a multivariate Cox regression model was used to calculate the hazard ratio (HR) and its 95% confidence interval (95% CI) to assess the impact of gender differences on survival benefit. Finally, subgroup analyses were conducted based on the aforementioned variables to calculate the HR and its 95% CI for each subgroup, identifying specific beneficial patient populations. All statistical analyses were performed using R software (version 4.3.1), and a P-value of < 0.05 was considered statistically significant.

## Results

### Baseline data

After screening according to the predefined inclusion and exclusion criteria, a total of 715 patients were included in the study, with 499 males and 216 females. The specific characteristics of both groups are summarized in Table 1. Regarding age, 56.94% of female patients were under 70 years old, compared to 47.09% of male patients. Tumors were more commonly located in the right lung, with the incidence of right lung malignancies being 57.87% in males and 54.51% in females. In terms of T stage, T2 and T4 stages were most common in the female group, each accounting for 32.41%, while the T4 stage was most common in the male group (43.69%). The N2 stage was the most prevalent in both male (53.51%) and female (50.46%) groups. The proportion of patients in the M1 stage was 55.11% in the male group and 62.96% in the female group. Regarding distant metastasis, bone metastasis was the most common in both male (21.44%) and female (31.48%) groups. In terms of pathological type, adenocarcinoma was the most common in females (93.52%), significantly higher than the proportion in males (57.31%). The EGFR mutation rate in the female group was 56.48%, notably higher than that in the male group (15.43%). With respect to treatment, the female group was more likely to receive EGFR-TKI therapy (59.26%), while only 17.64% of male patients received EGFR-TKI treatment. Additionally, 41.08% of male patients received ICIs therapy, while only 18.52% of female patients received ICIs therapy. Overall, statistically significant differences were observed in the distribution of age, AJCC T stage, bone metastasis, pathological type, EGFR mutation status, surgery, chemotherapy, radiotherapy, EGFR-TKI treatment, and ICIs treatment between the male and female groups (*P* < 0.05).

**Table 1:** Baseline characteristics of patients before PSM

Variable	Total (*n* = 715)	Female (*n* = 216)	Male (*n* = 499)	Statistic	*P*	SMD
Age, n (%)				χ²=5.851	0.016	
<70 years	358 (50.07)	123 (56.94)	235 (47.09)			−0.197
≥70 years	357 (49.93)	93 (43.06)	264 (52.91)			0.197
Laterality, n (%)				χ²=0.690	0.406	
Left	318 (44.48)	91 (42.13)	227 (45.49)			0.068
Right	397 (55.52)	125 (57.87)	272 (54.51)			−0.068
AJCC T, n (%)				χ²=20.712	< 0.001	
T1	96 (13.43)	42 (19.44)	54 (10.82)			−0.278
T2	187 (26.15)	70 (32.41)	117 (23.45)			−0.211
T3	144 (20.14)	34 (15.74)	110 (22.04)			0.152
T4	288 (40.28)	70 (32.41)	218 (43.69)			0.227
AJCC N, n (%)				χ²=6.174	0.103	
N0	167 (23.36)	60 (27.78)	107 (21.44)			−0.154
N1	53 (7.41)	19 (8.80)	34 (6.81)			−0.079
N2	376 (52.59)	109 (50.46)	267 (53.51)			0.061
N3	119 (16.64)	28 (12.96)	91 (18.24)			0.137
AJCC M, n (%)				χ²=3.804	0.051	
M0	304 (42.52)	80 (37.04)	224 (44.89)			0.158
M1	411 (57.48)	136 (62.96)	275 (55.11)			−0.158
Bone metastasis, n (%)				χ²=8.218	0.004	
No	540 (75.52)	148 (68.52)	392 (78.56)			0.245
Yes	175 (24.48)	68 (31.48)	107 (21.44)			−0.245
Brain metastasis, n (%)				χ²=3.358	0.067	
No	597 (83.5)	172 (79.63)	425 (85.17)			0.156
Yes	118 (16.5)	44 (20.37)	74 (14.83)			−0.156
Lung metastasis, n (%)				χ²=1.040	0.308	
No	579 (80.98)	170 (78.70)	409 (81.96)			0.085
Yes	136 (19.02)	46 (21.30)	90 (18.04)			−0.085
Liver metastasis, n (%)				χ²=2.807	0.094	
No	666 (93.15)	196 (90.74)	470 (94.19)			0.147
Yes	49 (6.85)	20 (9.26)	29 (5.81)			−0.147
Pathological pattern, n (%)				χ²=91.185	< 0.001	
Adenocarcinoma	488 (68.25)	202 (93.52)	286 (57.31)			−0.732
squamous carcinoma	227 (31.75)	14 (6.48)	213 (42.69)			0.732
EGFR, n (%)				χ²=126.473	< 0.001	
No	516 (72.17)	94 (43.52)	422 (84.57)			1.136
Yes	199 (27.83)	122 (56.48)	77 (15.43)			−1.136
Surgery, n (%)				χ²=6.784	0.009	
No	557 (77.9)	155 (71.76)	402 (80.56)			0.222
Yes	158 (22.1)	61 (28.24)	97 (19.44)			−0.222
Chemotherapy, n (%)				χ²=19.286	< 0.001	
No	231 (32.31)	95 (43.98)	136 (27.25)			−0.376
Yes	484 (67.69)	121 (56.02)	363 (72.75)			0.376
Radiation, n (%)				χ²=6.695	0.010	
No	674 (94.27)	211 (97.69)	463 (92.79)			−0.189
Yes	41 (5.73)	5 (2.31)	36 (7.21)			0.189
AAT, n (%)				χ²=0.001	0.982	
No	410 (57.34)	124 (57.41)	286 (57.31)			−0.002
Yes	305 (42.66)	92 (42.59)	213 (42.69)			0.002
EGFR TKI, n (%)				χ²=123.878	< 0.001	
No	499 (69.79)	88 (40.74)	411 (82.36)			1.092
Yes	216 (30.21)	128 (59.26)	88 (17.64)			−1.092
ICIs, n (%)				χ²=34.073	< 0.001	
No	470 (65.73)	176 (81.48)	294 (58.92)			−0.459
Yes	245 (34.27)	40 (18.52)	205 (41.08)			0.459

*EGFR* Epidermal Growth Factor Receptor, *AAT* Anti-angiogenesis targeted (Including bevacizumab, anlotinib and recombinant human endostatin),* EGFR TKI* Epidermal Growth Factor Receptor Tyrosine Kinase Inhibitor, *ICIs* immune checkpoint inhibitors(Including PD-1 monoclonal antibody and PD-L1 monoclonal antibody)

### PSM between the two groups

**Fig. 2:**
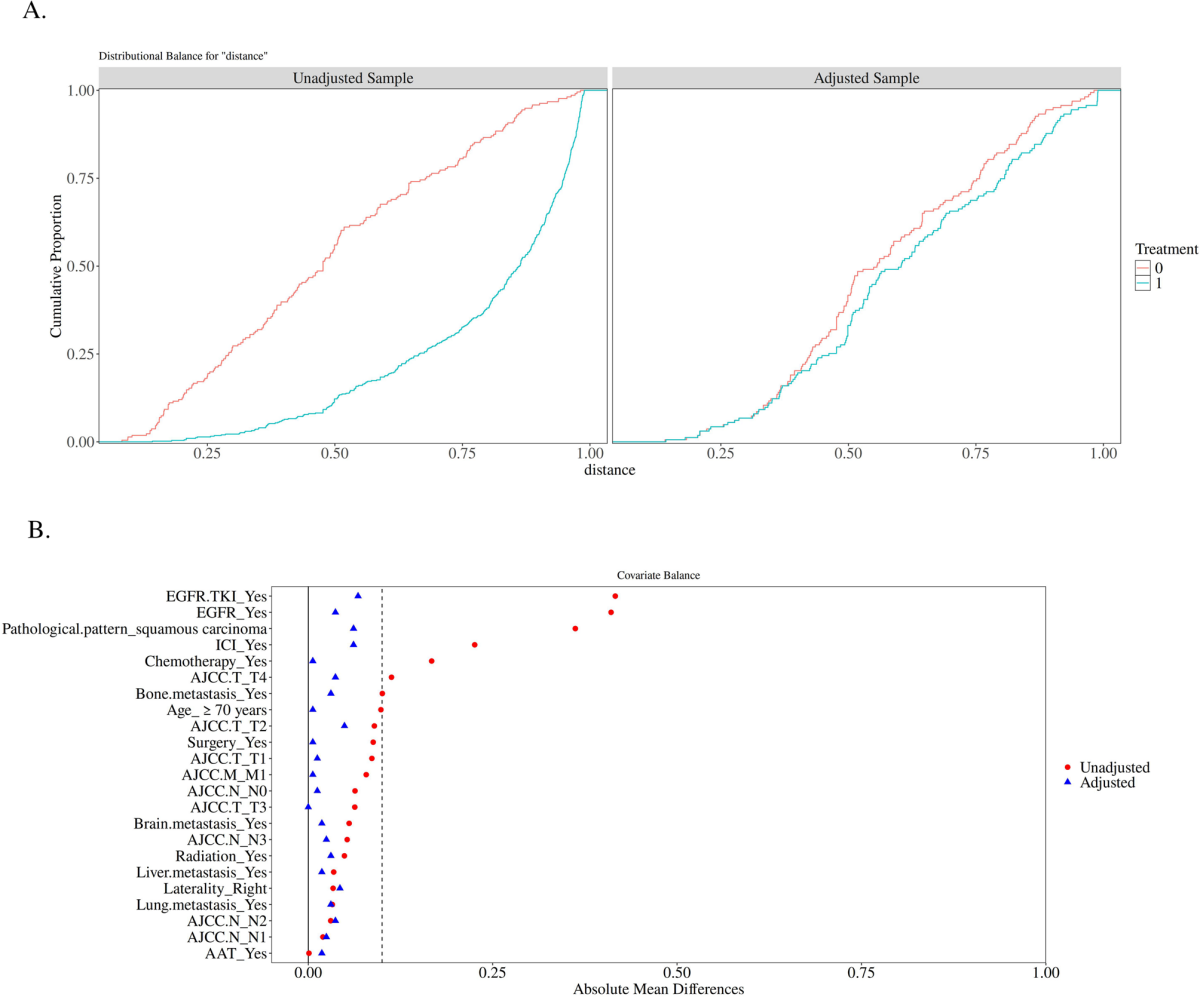
**A** shows the cumulative proportions of the two groups; **B** shows the absolute standardized mean of the two groups(“0” represents the male patient group, and “1” represents the female patient group)

Given the baseline imbalance between the two patient groups, which could introduce bias into the study results, we applied the PSM method to mitigate the impact of inter-group imbalance. Propensity scores(PS) were calculated using a Logistic regression model that included variables such as age, tumor location, T stage, N stage, M stage, distant metastasis (bone, brain, liver, lung), pathological type, EGFR mutation status, surgical treatment, chemotherapy, radiotherapy, AAT therapy, EGFR-TKI treatment, and ICIs treatment. A 1:1 match was performed using the MatchIt package in R software, with the caliper set at 0.25 times the standard deviation of the propensity score. After matching, the balance of baseline characteristics was assessed using the standardized mean difference (SMD), with an SMD value of less than 0.2 indicating balance. Ultimately, 326 patients were successfully matched, and no statistically significant differences in baseline characteristics were observed between the two groups after matching (*P* > 0.05) (Table 2). The matching results are shown in Fig. 2: Panel A demonstrates that the cumulative proportion curves for both groups closely overlap, indicating successful matching, while Panel B shows that the absolute standardized differences for both groups after matching are centered around 0, indicating the elimination of systemic differences.

**Table 2:** Baseline characteristics of patients after PSM

Variable	Total (*n* = 326)	Female (*n* = 163)	Male (*n* = 163)	Statistic	*P*	SMD
Age, n (%)				χ²=0.012	0.911	
<70 years	177 (54.29)	89 (54.60)	88 (53.99)			−0.012
≥70 years	149 (45.71)	74 (45.40)	75 (46.01)			0.012
Laterality, n (%)				χ²=0.610	0.435	
Left	143 (43.87)	68 (41.72)	75 (46.01)			0.086
Right	183 (56.13)	95 (58.28)	88 (53.99)			−0.086
AJCC T, n (%)				χ²=1.083	0.781	
T1	56 (17.18)	29 (17.79)	27 (16.56)			−0.033
T2	90 (27.61)	41 (25.15)	49 (30.06)			0.107
T3	60 (18.4)	30 (18.40)	30 (18.40)			0.000
T4	120 (36.81)	63 (38.65)	57 (34.97)			−0.077
AJCC N, n (%)				χ²=1.290	0.732	
N0	86 (26.38)	44 (26.99)	42 (25.77)			−0.028
N1	22 (6.75)	9 (5.52)	13 (7.98)			0.091
N2	164 (50.31)	85 (52.15)	79 (48.47)			−0.074
N3	54 (16.56)	25 (15.34)	29 (17.79)			0.064
AJCC M, n (%)				χ²=0.013	0.908	
M0	115 (35.28)	57 (34.97)	58 (35.58)			0.013
M1	211 (64.72)	106 (65.03)	105 (64.42)			−0.013
Bone metastasis, n (%)				χ²=0.371	0.542	
No	231 (70.86)	118 (72.39)	113 (69.33)			−0.067
Yes	95 (29.14)	45 (27.61)	50 (30.67)			0.067
Brain metastasis, n (%)				χ²=0.162	0.687	
No	255 (78.22)	129 (79.14)	126 (77.30)			−0.044
Yes	71 (21.78)	34 (20.86)	37 (22.70)			0.044
Lung metastasis, n (%)				χ²=0.450	0.502	
No	255 (78.22)	125 (76.69)	130 (79.75)			0.076
Yes	71 (21.78)	38 (23.31)	33 (20.25)			−0.076
Liver metastasis, n (%)				χ²=0.503	0.478	
No	307 (94.17)	152 (93.25)	155 (95.09)			0.085
Yes	19 (5.83)	11 (6.75)	8 (4.91)			−0.085
Pathological pattern, n (%)				χ²=2.979	0.084	
Adenocarcinoma	288 (88.34)	149 (91.41)	139 (85.28)			−0.173
squamous carcinoma	38 (11.66)	14 (8.59)	24 (14.72)			0.173
EGFR, n (%)				χ²=0.456	0.499	
No	192 (58.9)	93 (57.06)	99 (60.74)			0.075
Yes	134 (41.1)	70 (42.94)	64 (39.26)			−0.075
Surgery, n (%)				χ²=0.016	0.898	
No	245 (75.15)	123 (75.46)	122 (74.85)			−0.014
Yes	81 (24.85)	40 (24.54)	41 (25.15)			0.014
Chemotherapy, n (%)				χ²=0.013	0.910	
No	133 (40.8)	67 (41.10)	66 (40.49)			−0.012
Yes	193 (59.2)	96 (58.90)	97 (59.51)			0.012
Radiation, n (%)				χ²=2.003	0.157	
No	313 (96.01)	159 (97.55)	154 (94.48)			−0.134
Yes	13 (3.99)	4 (2.45)	9 (5.52)			0.134
AAT, n (%)				χ²=0.113	0.736	
No	189 (57.98)	93 (57.06)	96 (58.90)			0.037
Yes	137 (42.02)	70 (42.94)	67 (41.10)			−0.037
EGFR TKI, n (%)				χ²=1.512	0.219	
No	185 (56.75)	87 (53.37)	98 (60.12)			0.138
Yes	141 (43.25)	76 (46.63)	65 (39.88)			−0.138
ICIs, n (%)				χ²=1.716	0.190	
No	250 (76.69)	130 (79.75)	120 (73.62)			−0.139
Yes	76 (23.31)	33 (20.25)	43 (26.38)			0.139

*EGFR* Epidermal Growth Factor Receptor, *AAT* Anti-angiogenesis targeted (Including bevacizumab, anlotinib and recombinant human endostatin), *EGFR TKI* Epidermal Growth Factor Receptor Tyrosine Kinase Inhibitor, *ICIs* Immune checkpoint inhibitors (Including PD-1 monoclonal antibody and PD-L1 monoclonal antibody)

### Kaplan-Meier survival analysis

**Fig. 3:**
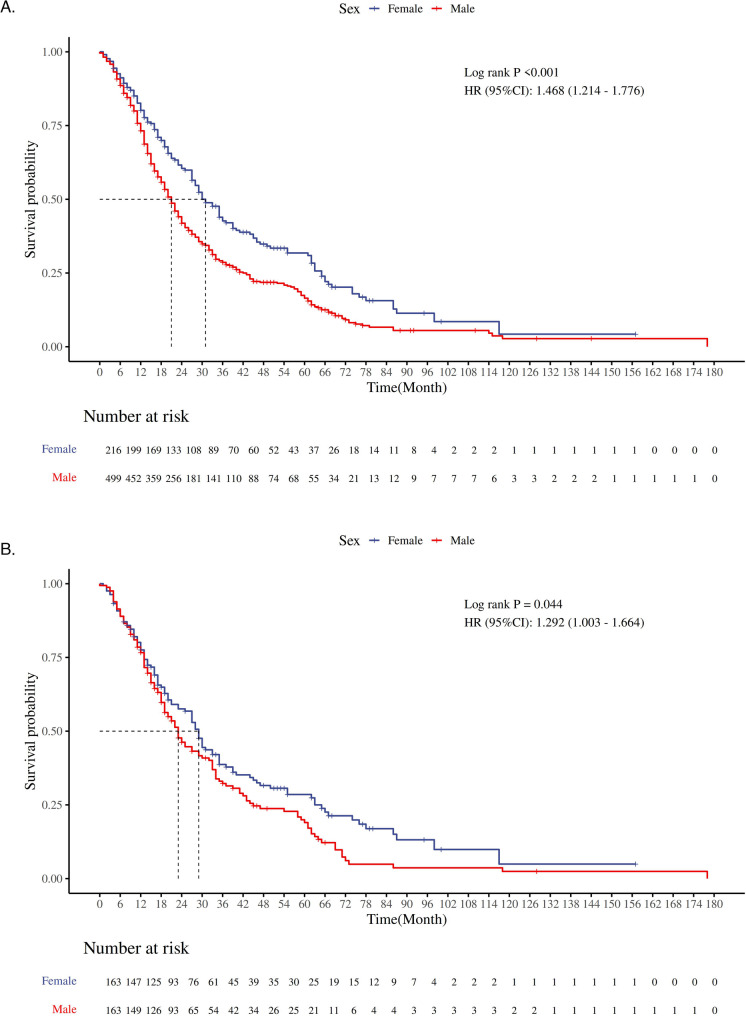
**A** shows the Kaplan-Meier survival curves for OS time in the two groups before PSM; **B** shows the Kaplan-Meier survival curves for OS time in the two groups after PSM

**Fig. 4:**
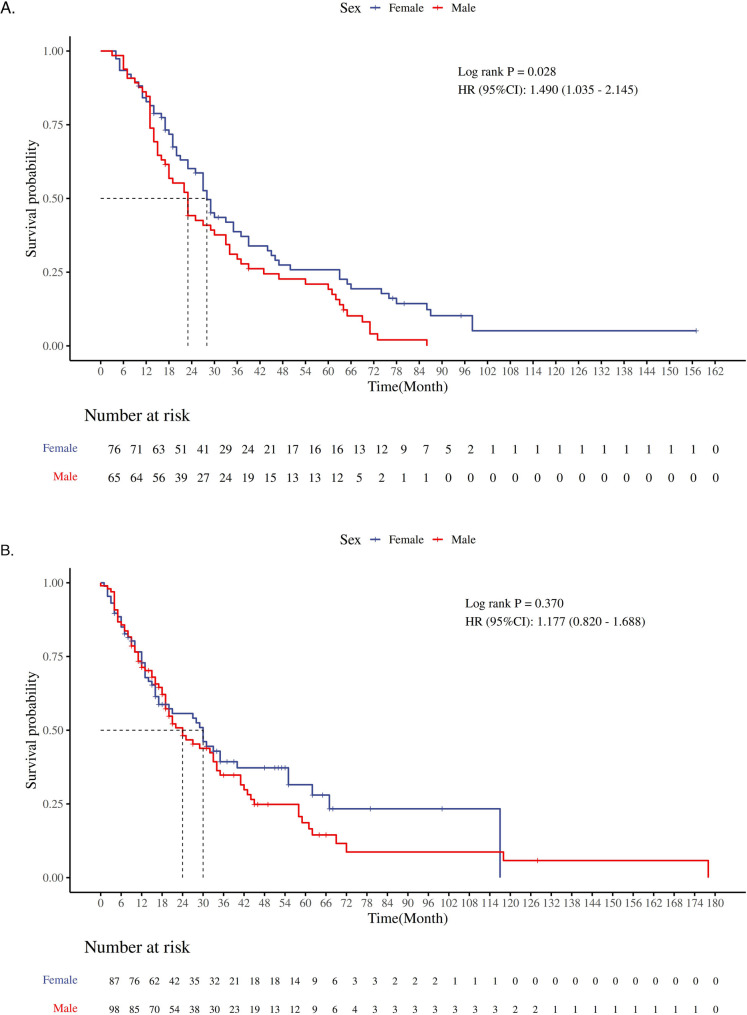
**A** represents the Kaplan-Meier survival curve of OS time for patients receiving EGFR-TKI treatment; **B** represents the Kaplan-Meier survival curve of OS time for patients not receiving EGFR-TKI treatment

**Fig. 5:**
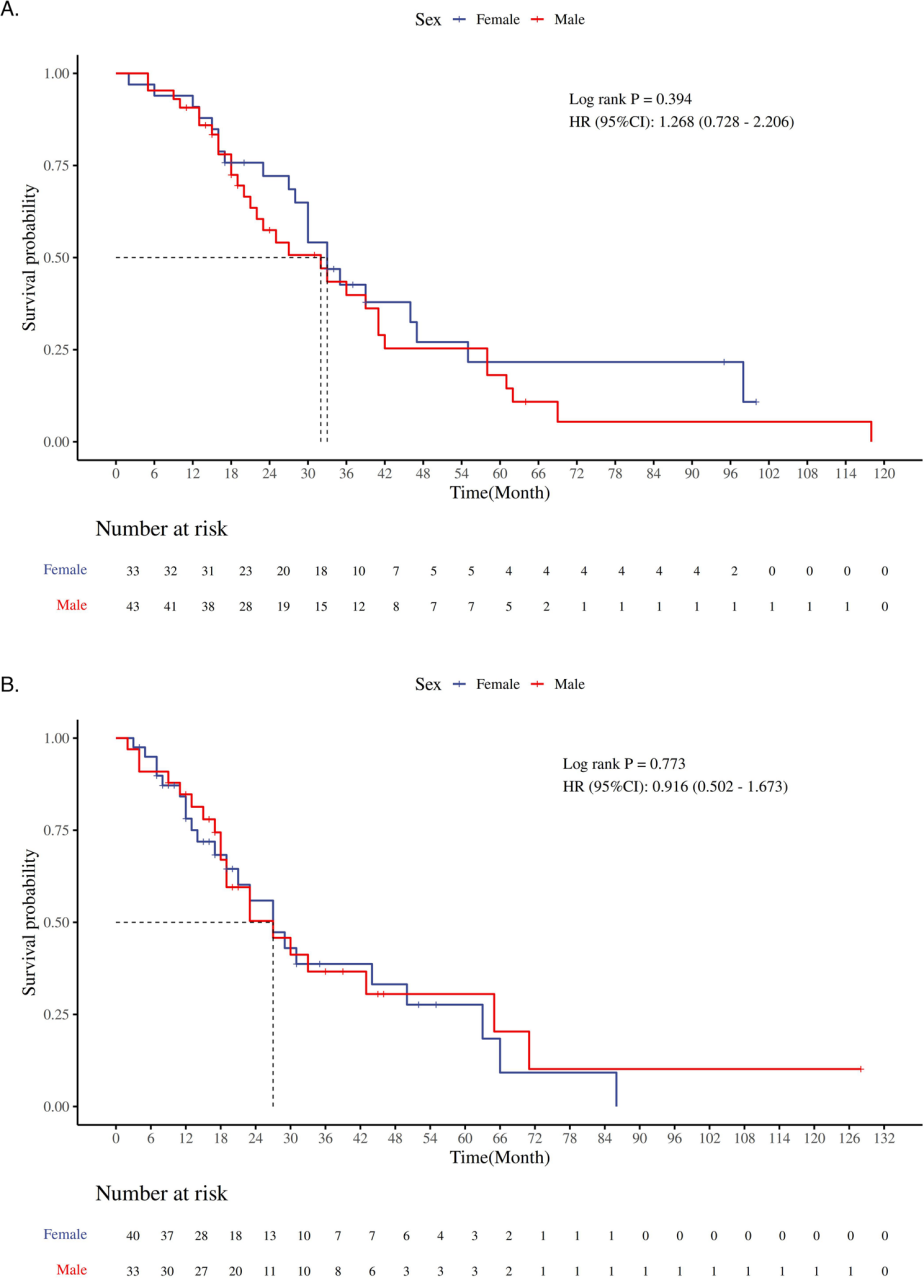
**A** represents the Kaplan-Meier survival curve of OS time for patients who received ICIs treatment; **B** represents the Kaplan-Meier survival curve of OS time for patients who did not receive ICIs treatment

Kaplan-Meier survival analysis showed that in the unmatched dataset, the mortality risk for male patients was 46.8% higher than that for female patients (HR = 1.468, 95% CI 1.214–1.776, *P* < 0.001). Given the baseline imbalance between the two groups, this result may be biased. After PSM, a repeated Kaplan-Meier analysis revealed that the median survival times for the male and female groups were 23 months and 29 months, respectively. The mortality risk for male patients was 29.2% higher than that for female patients (HR = 1.292, 95% CI 1.003–1.664, *P* = 0.044), and the difference was statistically significant (Fig. 3). Further stratified analysis of patients treated with EGFR-TKI and ICIs showed that, among EGFR-TKI-treated patients, the mortality risk for males was 49% higher than that for females (HR = 1.490, 95% CI 1.035–2.145, *P* = 0.028), while in patients not treated with EGFR-TKI, no statistically significant survival differences were observed between the two groups (HR = 1.177, 95% CI 0.820–1.688, *P* = 0.370) (Fig. 4). For ICIs-treated patients, no statistically significant survival differences were observed between the two groups, regardless of whether they received ICIs treatment (ICIs-treated: HR = 1.268, 95% CI 0.728–2.206, *P* = 0.394; non-ICIs-treated: HR = 0.916, 95% CI 0.502–1.673, *P* = 0.773) (Fig. 5).

### Cause of death analysis

**Fig. 6:**
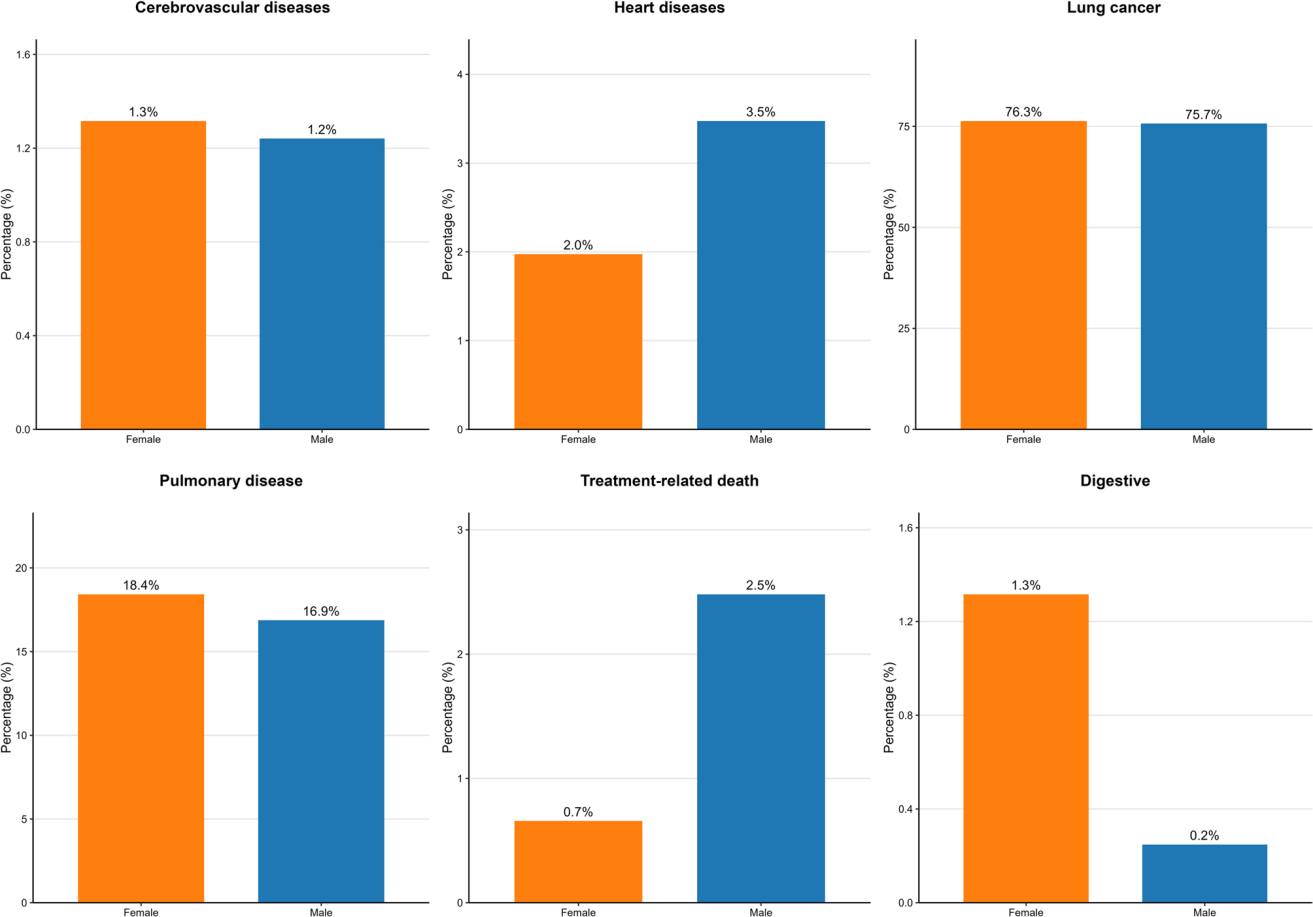
The causes of death of the two groups of patients

Our study included a total of 715 patients, of whom 555 patients died (403 males and 152 females). The distribution of different causes of death between male and female patients was compared using the Chi-square test or Fisher’s exact test. The results showed that no statistically significant differences were observed in the distribution of causes of death between genders (*P* > 0.05). Specifically, lung cancer-related death had the highest proportion (male 75.68% vs. female 76.32%, *P* = 0.871), suggesting that lung cancer remains the leading cause of death, with no gender difference in its distribution. The second most common cause of death was lung diseases, with no significant gender difference observed (male 16.87% vs. female 18.42%, *P* = 0.873). There were no significant gender differences in the distribution of deaths due to cerebrovascular diseases (male 1.24% vs. female 1.32%, *P* = 0.665) or heart disease (male 3.47% vs. female 1.97%, *P* = 0.605). Other causes of death, such as treatment-related death (male 2.48% vs. female 0.66%, *P* = 0.670) and digestive system diseases (male 0.25% vs. female 1.32%, *P* = 0.183), also showed no significant gender differences. Detailed results are shown in Table 3; Fig. 6.

**Table 3:** Mortality analysis

Variables	Total (*n* = 555)	Female (*n* = 152)	Male (*n* = 403)	Statistic	*P*
Cerebrovascular diseases, n(%)				χ²=0.186	0.665
No	548 (98.74)	150 (98.68)	398 (98.76)		
Yes	7 (1.26)	2 (1.32)	5 (1.24)		
Heart diseases, n(%)				χ²=0.268	0.605
No	538 (96.94)	149 (98.03)	389 (96.53)		
Yes	17 (3.06)	3 (1.97)	14 (3.47)		
Lung cancer, n(%)				χ²=0.026	0.871
No	134 (24.14)	36 (23.68)	98 (24.32)		
Yes	421 (75.86)	116 (76.32)	305 (75.68)		
Pulmonary disease, n(%)				χ²=0.025	0.873
No	459 (82.70)	124 (81.58)	335 (83.13)		
Yes	96 (17.30)	28 (18.42)	68 (16.87)		
Treatment-related death, n(%)				χ²=0.181	0.670
No	544 (98.02)	151 (99.34)	393 (97.52)		
Yes	11 (1.98)	1 (0.66)	10 (2.48)		
Digestive, n(%)				-	0.183
No	552 (99.46)	150 (98.68)	402 (99.75)		
Yes	3 (0.54)	2 (1.32)	1 (0.25)		

χ²: Chi-square test, -: Fisher exact

### Univariate and multivariate Cox analysis

In this study, we used multivariate Cox regression analysis to directly assess potential prognostic factors affecting the OS of NSCLC patients. This method allows for the simultaneous consideration of interactions between multiple variables, avoiding biases that might arise from univariate analysis and thereby more accurately identifying independent prognostic factors. The multivariate analysis results showed that the mortality risk for male patients was significantly higher than that for female patients (HR = 1.31, 95% CI: 1.01–1.70, *P* = 0.046). AJCC T stage (T2: HR = 2.10, 95% CI: 1.25–3.54, *P* = 0.005; T3: HR = 2.20, 95% CI: 1.24–3.92, *P* = 0.007; T4: HR = 2.76, 95% CI: 1.59–4.79, *P* < 0.001), N stage (N1: HR = 2.21, 95% CI: 1.15–4.25, *P* = 0.017; N2: HR = 2.23, 95% CI: 1.44–3.45, *P* < 0.001; N3: HR = 2.33, 95% CI: 1.43–3.78, *P* < 0.001), and M stage (M1: HR = 3.19, 95% CI: 2.00–5.11.00.11, *P* < 0.001) were all identified as independent prognostic factors affecting OS. Furthermore, patients who received chemotherapy (HR = 0.58, 95% CI: 0.42–0.79, *P* < 0.001) and AAT therapy (HR = 0.67, 95% CI: 0.49–0.91, *P* = 0.012) had a significantly reduced risk of death. Other factors, including age, tumor location, bone metastasis, brain metastasis, lung metastasis, liver metastasis, pathological type, EGFR mutation status, surgery, radiotherapy, EGFR-TKI treatment, and ICIs treatment, did not show a significant prognostic impact on OS in the multivariate analysis (*P* > 0.05) (Table 4).

**Table 4:** Multivariate COX regression analyses of OS

Variables	β	S.E	Z	*P*	HR (95%CI)
Age					
<70 years					Reference
≥70 years	−0.11	0.15	−0.74	0.459	0.90 (0.67–1.20)
Sex					
Female					Reference
Male	0.27	0.13	2.00	0.046	1.31 (1.01–1.70)
Laterality					
Left					Reference
Right	−0.13	0.14	−0.96	0.340	0.88 (0.67–1.15)
AJCC T					
T1					Reference
T2	0.74	0.27	2.79	0.005	2.10 (1.25–3.54)
T3	0.79	0.29	2.68	0.007	2.20 (1.24–3.92)
T4	1.01	0.28	3.60	< 0.001	2.76 (1.59–4.79)
AJCC N					
N0					Reference
N1	0.79	0.33	2.39	0.017	2.21 (1.15–4.25)
N2	0.80	0.22	3.60	< 0.001	2.23 (1.44–3.45)
N3	0.85	0.25	3.42	< 0.001	2.33 (1.43–3.78)
AJCC M					
M0					Reference
M1	1.16	0.24	4.84	< 0.001	3.19 (2.00–5.11.00.11)
Bone metastasis					
No					Reference
Yes	0.03	0.16	0.21	0.830	1.03 (0.76–1.41)
Brain metastasis					
No					Reference
Yes	0.12	0.17	0.71	0.476	1.13 (0.81–1.57)
Lung metastasis					
No					Reference
Yes	−0.23	0.18	−1.28	0.201	0.80 (0.56–1.13)
Liver metastasis					
No					Reference
Yes	0.27	0.28	0.99	0.324	1.31 (0.76–2.27)
Pathological pattern					
Adenocarcinoma					Reference
squamous carcinoma	0.17	0.25	0.69	0.490	1.19 (0.73–1.93)
EGFR					
No					Reference
Yes	−0.17	0.30	−0.58	0.559	0.84 (0.47–1.51)
Surgery					
No					Reference
Yes	−0.30	0.28	−1.10	0.270	0.74 (0.43–1.27)
Chemotherapy					
No					Reference
Yes	−0.55	0.16	−3.41	< 0.001	0.58 (0.42–0.79)
Radiation					
No					Reference
Yes	−0.17	0.38	−0.46	0.645	0.84 (0.40–1.76)
AAT					
No					Reference
Yes	−0.40	0.16	−2.52	0.012	0.67 (0.49–0.91)
EGFR TKI					
No					Reference
Yes	−0.50	0.30	−1.69	0.091	0.60 (0.34–1.08)
ICIs					
No					Reference
Yes	−0.34	0.19	−1.80	0.072	0.71 (0.49–1.03)

*HR* Hazard Ratio, *CI* Confidence Interval, *EGFR* Epidermal Growth Factor Receptor, *AAT* Anti-angiogenesis targeted(Including bevacizumab, anlotinib and recombinant human endostatin), *EGFR TKI* Epidermal Growth Factor Receptor Tyrosine Kinase Inhibitor, *ICIs* immune checkpoint inhibitors (Including PD-1 monoclonal antibody and PD-L1 monoclonal antibody)

### Subgroup analysis

**Fig. 7:**
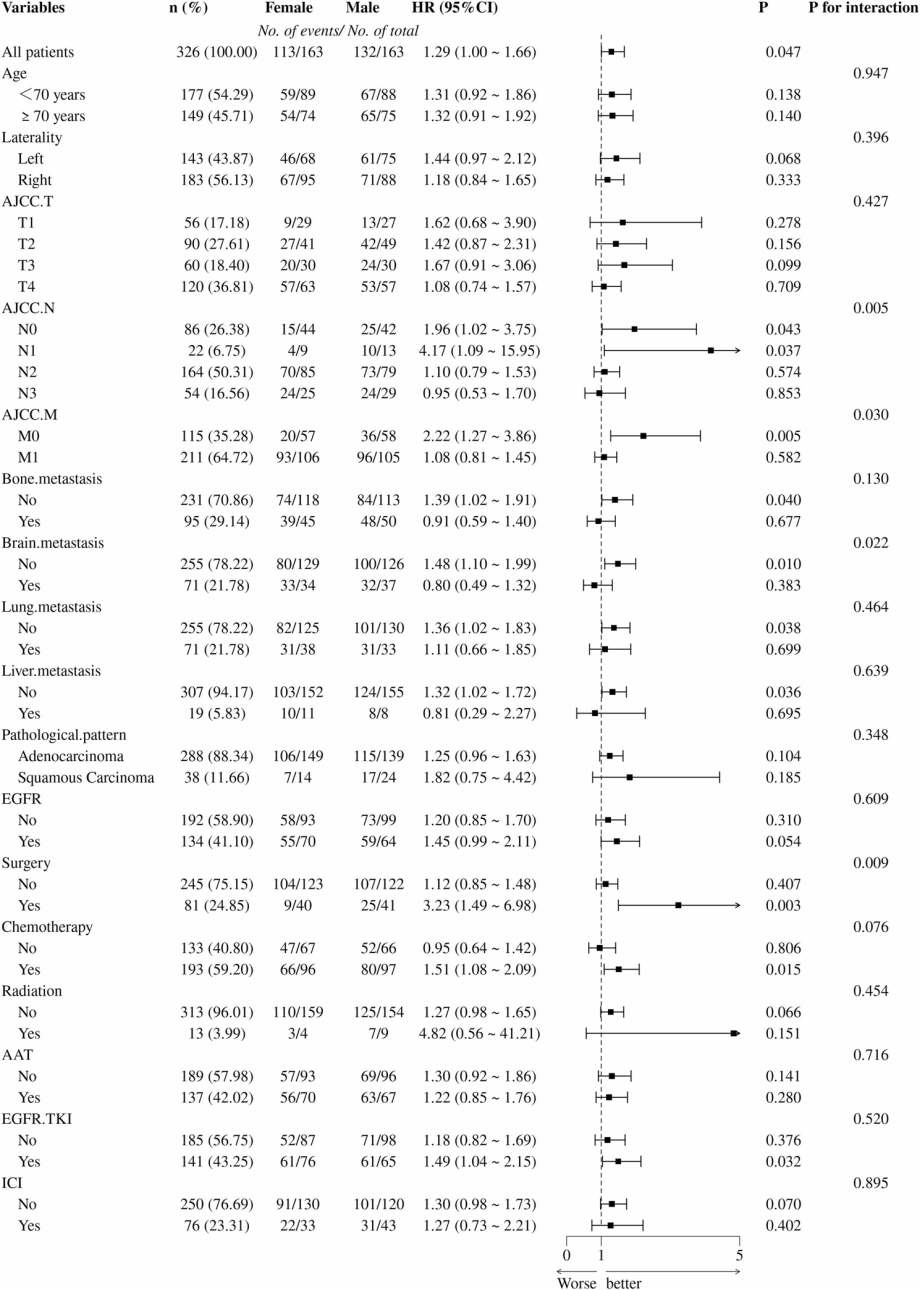
The subgroup analysis results of OS

In this study, we conducted a subgroup analysis to explore gender differences in NSCLC patients and assess specific prognostic benefits in each subgroup. The subgroup analysis revealed significant heterogeneity in the impact of gender on NSCLC prognosis under specific clinical stages and treatment modalities. First, the interaction P-value for AJCC N stage was 0.005. In the N0 stage (HR = 1.96, *P* = 0.043) and N1 stage (HR = 4.17, *P* = 0.037) subgroups, male patients had worse prognoses, while no gender differences were observed in the N2 and N3 stages. Second, the interaction P-value for AJCC M stage was 0.030. In the M0 stage subgroup, male patients showed a significantly higher mortality risk (HR = 2.22, *P* = 0.005), but no gender differences were found in the M1 stage. Furthermore, there was a significant interaction P-value for brain metastasis status (*P* = 0.022). Specifically, male patients without brain metastasis had a higher mortality risk (HR = 1.48, *P* = 0.010), whereas no gender differences were observed in patients with brain metastasis. Finally, a significant interaction P-value was observed in the surgical treatment subgroup (*P* = 0.009). Male patients who underwent surgery had significantly worse prognoses than females (HR = 3.23, *P* = 0.003), while no significant differences were observed in the non-surgical subgroup. Although the interaction in the chemotherapy subgroup did not reach statistical significance (*P* = 0.076), male patients who received chemotherapy still showed an increased risk trend (HR = 1.51, 95% CI: 1.08–2.09). No gender-related prognostic heterogeneity was observed in other subgroups, including age, tumor location, T stage, pathological type, bone/lung/liver metastasis, chemotherapy, radiotherapy, AAT therapy, EGFR-TKI treatment, or ICIs treatment (*P* > 0.05). These results suggest that male patients have significantly worse prognoses than females in the early stages of lymph node metastasis (N0-1), without distant metastasis (M0), without brain metastasis, or when undergoing surgery (Fig. 7).

## Discussion

This study utilized real-world cohort data and applied PSM to explore the prognostic differences in NSCLC patients. The results showed that the OS of male patients was significantly lower than that of female patients (HR = 1.292, 95% CI: 1.003–1.664, *P* = 0.044), suggesting that gender may be an independent factor influencing NSCLC prognosis. Further multivariate Cox regression analysis confirmed this conclusion, with male patients exhibiting a 31% higher mortality risk than female patients (HR = 1.31, 95% CI: 1.01–1.70, *P* = 0.046), independent of AJCC staging and treatment modalities. In different treatment subgroups, gender differences showed some heterogeneity. In particular, in the EGFR-TKI treatment subgroup, male patients had a higher mortality risk (HR = 1.490, 95% CI: 1.035–2.145, *P* = 0.028), while no statistically significant gender differences were observed in the ICIs treatment subgroup (*P* > 0.05). Subgroup analysis further revealed the heterogeneity of gender’s prognostic effect. In patients with early lymph node metastasis (N0-1), no distant metastasis (M0), no brain metastasis, and those who underwent surgery, male patients had a significantly higher mortality risk than females (interaction *P* < 0.05). These results underscore the importance of considering gender in the individualized treatment of NSCLC, which aids in more accurately assessing prognosis and making treatment decisions. The findings of this study suggest that gender has a significant impact on NSCLC prognosis, particularly under specific clinical stages and treatment modalities. This discovery indicates that gender should be incorporated into treatment decision-making in clinical practice.

When placing the results of this study in the context of previous research, we found that our findings align with current trends in studies on gender differences in NSCLC, while also presenting some differences. On one hand, this study confirms the impact of gender on NSCLC prognosis, consistent with an increasing number of studies in recent years that highlight gender differences in the biological behavior of lung cancer and treatment responses [12–14]. For instance, some studies have suggested that women may have a better prognosis in NSCLC, which is linked to factors such as hormone levels, gene expression patterns, and differing sensitivities to treatment. However, this study further reveals that gender differences are more pronounced under specific treatment modalities, particularly in the EGFR-TKI treatment subgroup (HR = 1.490, 95% CI: 1.035–2.145, *P* = 0.028). This finding may be associated with differences in EGFR mutation rates between the sexes. Male patients tend to have a higher smoking rate than females, and smoking can lead to alterations in gene expression patterns, which in turn affects the propensity for EGFR mutations. As a result, male patients generally exhibit a lower EGFR mutation rate, while the higher EGFR mutation rate in female patients makes them more likely to receive EGFR-TKI treatment [15, 16]. This could, to some extent, explain the prognostic differences between male and female patients in this treatment subgroup. Due to the limitations of the case system at this center, this study was unable to obtain specific smoking data from the patients. Nevertheless, smoking is widely acknowledged as a significant prognostic factor in NSCLC, as it is linked not only to a poor response to EGFR-TKI treatment but also to adverse outcomes following surgical intervention. Previous studies have indicated that smokers are at a higher risk of complications both pre- and postoperatively compared to non-smokers, contributing to a poorer overall prognosis [17]. The subgroup analysis of this study revealed that the mortality risk in male patients undergoing surgery was significantly higher than that in females, with a higher smoking rate among males potentially contributing to this difference. Furthermore, smoking influences the efficacy of chemotherapy by inducing cytochrome P450 enzyme activity, which accelerates the metabolism of various chemotherapy drugs, resulting in decreased drug concentrations in the body and, consequently, reduced treatment efficacy [18, 19]. A multicenter study conducted in the UK reported that the 2-year survival rate for patients who quit smoking after diagnosis was 0.45 (95%CI 0.37–0.53), while for those who continued smoking it was 0.32 (95%CI 0.28–0.36), with the difference being statistically significant (*P* < 0.01) [20]. This finding further substantiates the detrimental impact of smoking on the efficacy of EGFR-TKI, surgery, and chemotherapy. The baseline characteristics of this study revealed that the proportion of male patients with squamous cell lung cancer was significantly higher than that of female patients (42.69% vs. 6.48%), with smoking identified as a major risk factor for squamous cell lung cancer. Although this study did not calculate the smoking rate by gender, the results above indirectly suggest that the smoking rate among male patients may be higher, which could partly contribute to reduced sensitivity to EGFR-TKI, surgery, and chemotherapy, thereby leading to a relatively poorer prognosis in male patients. It is important to note that the specific effects of smoking and its underlying mechanisms remain to be elucidated in future studies, which should include the systematic collection of smoking data.Additionally, some studies suggest that estrogen can enhance the efficacy of EGFR-TKI through interactions with the EGFR receptor [21]. The combined effect of these factors may have contributed to better prognosis in female patients undergoing EGFR-TKI treatment. Our finding resonates with earlier studies that suggested targeted therapy may be more effective in female patients. For example, in the FLAURA trial [10], the HR for female patients was 0.40, reducing the risk of death by 18% compared to male patients. This viewpoint was also confirmed in adjuvant therapy for stage IB-IIIA NSCLC, where the OS HR for male patients was 0.51 (95% CI: 0.32–0.81) and for female patients, the OS HR was 0.47 (95% CI: 0.29–0.76) [22]. Therefore, whether in adjuvant therapy for NSCLC or in first-line treatment for advanced stages, female patients exhibit better therapeutic outcomes than male patients, consistent with the findings of previous studies. However, by applying PSM analysis, this study reduces the impact of potential confounding factors and more precisely quantifies the effect of gender differences on prognosis.

In this study, the multivariable Cox regression analysis revealed that EGFR-TKI treatment was not an independent prognostic factor for OS (*P* > 0.05). However, stratified Kaplan-Meier analysis revealed a significant gender difference in the subgroup of patients receiving EGFR-TKI treatment, with the mortality risk for male patients 49% higher than that for female patients (HR = 1.490, 95% CI 1.035–2.145, *P* = 0.028). This discrepancy may arise from the characteristics of the two statistical methods. The multivariable Cox analysis offers a comprehensive evaluation of multiple prognostic factors, considering their interactions. In this study, the effect of EGFR-TKI may have been obscured by other stronger prognostic factors, which could explain the lack of statistical significance. In contrast, Kaplan-Meier analysis, a descriptive method, offers an intuitive depiction of survival differences between specific subgroups, and when stratified by EGFR-TKI treatment, it more clearly highlights the impact of gender on prognosis. These findings suggest that relying solely on a single statistical method may not be adequate when assessing treatment effects. Cox regression is suitable for multivariable adjustment but may overlook subgroup-specific differences, while Kaplan-Meier analysis intuitively reflects survival curve differences but struggles to control for confounding factors simultaneously. Therefore, integrating multiple statistical methods for a comprehensive interpretation is preferable. By integrating two statistical methods, we found that male patients in the EGFR-TKI treatment group had a poorer prognosis, indicating that gender differences in NSCLC patients should be considered, particularly for male patients receiving EGFR-TKI treatment or those at specific clinical stages, as their prognosis may be worse. When developing treatment plans, clinicians should adopt individualized strategies, including multimodal therapies, enhanced follow-up and dynamic monitoring, and lifestyle interventions, such as smoking cessation, to improve prognosis.

In addition, in our study, the ICIs treatment subgroup did not show statistically significant gender differences. Several previous studies on ICIs treatment for NSCLC have shown that female patients had better progression-free survival(PFS) and OS compared to male patients [23–25]. Specifically, in the KEYNOTE-189 trial, the HR for male patients was 0.49, indicating a 51% reduction in the risk of death in male patients receiving Pembrolizumab plus Chemotherapy; whereas for female patients, the HR was 0.29, suggesting a 71% reduction in the risk of death, highlighting that female patients benefit more significantly in terms of OS [26]. However, in our study, neither the stratified survival analysis (ICIs treatment: HR = 1.268, 95% CI: 0.728–2.206, *P* = 0.394; no ICIs treatment: HR = 0.916, 95% CI: 0.502–1.673, *P* = 0.773) nor the subgroup analysis (interaction *P* = 0.895) observed any significant survival differences between male and female patients receiving ICIs treatment. This discrepancy may stem from differences in the patient populations included in various studies, variations in treatment regimens, and differences in data quality. For example, earlier studies often focused on specific types of NSCLC (such as adenocarcinoma or squamous cell carcinoma) or specific treatment stages (such as advanced or metastatic lung cancer), while our study included a broad patient population across various antitumor treatment modalities. This design may have impacted the observation of gender differences, but its strength lies in providing a more comprehensive reflection of real-world treatment responses, offering richer evidence-based support for clinical practice [27]. Furthermore, differences in the enrolled populations should not be overlooked. Previous studies primarily involved Caucasians from Europe and the United States, while all participants in this study were of Chinese descent. The impact of ethnic characteristics on the efficacy of ICIs treatment has increasingly become a topic of attention in recent research. Previous studies have suggested that ethnic differences may play a significant role in the efficacy of ICIs treatment and the incidence of adverse events. However, due to the lack of ethnic diversity in clinical trials and inconsistencies in reporting standards, our understanding of how ethnic characteristics precisely affect ICIs treatment efficacy remains limited [28, 29].The impact of statistical factors on the results should not be underestimated. In this study, the PSM method was employed to balance baseline characteristics. However, after matching, the sample size in the subgroup receiving ICIs treatment remained relatively small, potentially reducing the statistical power to detect significant gender differences. An insufficient sample size weakens the ability to detect differences, making it difficult to achieve statistical significance, even if a true difference exists. Future studies should increase sample size, prioritize the inclusion of participants from diverse backgrounds, and utilize standardized methods to report and analyze racial and other relevant factors to more comprehensively assess their potential impact on ICIs efficacy.

This study holds significant clinical implications by highlighting the role of gender factors in treatment decision-making for NSCLC, especially at particular clinical stages and treatment modalities. The results suggest that male patients have a significantly poorer prognosis compared to female patients when receiving EGFR-TKI treatment, possibly due to differences in EGFR mutation rates between genders and the potential modulatory effect of estrogen on EGFR-TKI efficacy. This finding implies that, in clinical practice, male patients undergoing EGFR-TKI treatment should be carefully assessed for prognostic risk, and consideration should be given to combining additional treatment modalities to optimize treatment outcomes. Moreover, this study suggests that male patients face a higher mortality risk in cases of early lymph node metastasis (N0-1), no distant metastasis (M0), no brain metastasis, and in those undergoing surgical treatment. This may be attributed to a lower sensitivity to treatment in male patients in these clinical scenarios. Therefore, when formulating treatment plans, more frequent monitoring and potentially more aggressive interventions should be considered for male patients to improve their prognosis. For instance, male patients undergoing surgery may require more frequent follow-up and monitoring to address their comparatively higher mortality risk. These findings offer valuable guidance for future research, encouraging further exploration of the molecular mechanisms underlying gender differences, such as examining gene expression profiles, hormone levels, and the immune microenvironment, to uncover the deeper reasons behind the influence of gender on treatment response. Furthermore, future research should explore methods to better incorporate gender-specific factors into treatment strategies to enhance patients’ OS and quality of life.

In this study, we examined the causes of death in male and female NSCLC patients. The results revealed that lung cancer was the leading cause of death for both genders, accounting for 76.32% and 75.68%, respectively, with no significant difference (*P* = 0.871). This finding suggests that most patients died as a result of the cancer itself, though it does not directly account for the shorter survival time observed in male patients. Nevertheless, this analysis offers valuable insights into the role of gender differences in disease progression and prognosis. First, this result eliminates potential interference from deaths due to other diseases in the overall survival analysis, allowing a more focused examination of the impact of lung cancer-related factors on prognosis. Second, this finding emphasizes the importance of standardized treatment and management of lung cancer in clinical practice, irrespective of gender, to improve overall survival outcomes. Notably, although the primary cause of death was the same for both genders, male patients had a shorter survival time, which may be attributed to various clinical and treatment factors. For instance, in specific treatment subgroups (e.g., EGFR-TKI treatment group), male patients exhibited worse prognoses, and in certain clinical stages (e.g., early-stage lymph node metastasis, absence of distant metastasis, no brain metastasis, or those undergoing surgery), male patients also had a higher mortality risk. These factors may interact and contribute to the shorter survival time observed in male patients. Therefore, although the analysis of causes of death did not directly explain the gender differences, its results provide important context for further exploration of the potential mechanisms behind the shorter survival in male patients, suggesting that a comprehensive consideration of multiple factors in clinical management is essential to optimize the prognosis of male NSCLC patients.

This study presents several innovations in the field of gender differences in NSCLC research. First, the data used in this study are derived from real-world settings, providing a more comprehensive and accurate representation of clinical situations. The conclusions drawn are more applicable to clinical practice, which is an advantage of real-world research [27, 30]. Additionally, we conducted a comprehensive analysis of the impact of gender on prognosis under various anti-tumor treatment modalities. This analysis included traditional surgery, chemotherapy, and radiotherapy, as well as the incorporation of targeted therapies involving driver gene mutations and ICIs therapy, which have been increasingly applied in recent years. This approach provides a more robust data set for evaluating gender differences. Second, this study employed a PSM method, effectively addressing imbalances in baseline characteristics and minimizing the influence of potential confounding factors, thereby enhancing the reliability and persuasiveness of the study’s findings [31]. Furthermore, the study conducted an in-depth subgroup analysis of specific clinical stages and treatment subgroups, revealing the heterogeneity of gender differences in various contexts and offering valuable insights for clinical individualized treatment. However, this study has several limitations. First, this study is retrospective. Although statistical methods such as PSM were employed to control for confounding factors, there may still be unidentified or inadequately addressed confounders that could impact the interpretation of the results. Second, the data in this study were derived from a single center with a relatively limited sample size, and the patients included were exclusively of Chinese ethnicity. This may limit the generalizability of the study’s findings and hinder direct application to other racial or regional populations. Furthermore, the follow-up period in this study was relatively short, and long-term survival data for some patients have not yet fully matured. This may influence the accurate assessment of long-term prognosis differences. Furthermore, this study did not investigate the underlying biological mechanisms contributing to gender differences, including variations in hormone levels, changes in gene expression patterns, and differences in the immune microenvironment. Lastly, the study did not incorporate specific tobacco use variables, such as the age of smoking onset, daily cigarette consumption, or smoking duration. Future research should gather detailed smoking status data to more accurately assess the impact of smoking on the prognosis of NSCLC patients. Future studies should also further investigate how smoking influences the efficacy of various treatment modalities and how smoking cessation interventions could enhance patient prognosis.Despite these limitations, the findings of this study offer new insights into gender differences in NSCLC research and provide important references for individualized treatment decisions in clinical practice. Given these limitations, future research should further validate these findings and explore the potential biological mechanisms underlying gender differences. Multi-center, prospective studies are recommended to validate the findings of this study and further explore the potential mechanisms underlying gender differences. Furthermore, future studies should focus on recruiting diverse populations and adopting standardized methods to report and analyze gender and other relevant factors, thereby enabling a more comprehensive assessment of their impact on treatment response in NSCLC.

## Conclusion

This study highlights the independent impact of gender on prognosis in NSCLC based on real-world data. Male patients exhibited shorter OS compared to female patients, particularly when receiving EGFR-TKI treatment or during specific clinical stages (e.g., early lymph node metastasis, no distant metastasis, no brain metastasis, or undergoing surgical treatment), where the prognosis for males was markedly worse. These findings imply that gender should be incorporated into individualized treatment decisions for NSCLC, especially in targeted therapies and surgical treatments, necessitating more detailed prognostic assessments for male patients. The results of this study lay the foundation for optimizing clinical treatment strategies. However, due to the retrospective design and the single-center sample, further validation through multi-center prospective studies is required, as well as an in-depth exploration of the biological mechanisms underlying gender differences.

## Data Availability

The datasets used and/or analyzed in this study are available from the corresponding author upon reasonable request.

## Abbreviations

NSCLC: Non-small cell lung cancer
SEER: Surveillance, Epidemiology, and End Results
ICIs: Immune checkpoint inhibitors
NIH: National Institutes of Health's
AAT: Anti-angiogenesis targeted
EGFR: Epidermal Growth Factor Receptor
TKI: Tyrosine Kinase Inhibitor
OS: Overall survival
PSM: Propensity score matching
HR: Hazard ratio
95% CI: 95% confidence interval
PS: Propensity scores
SMD: Standardized mean difference
PFS: Progression-free survival
